# Gelam Honey Attenuates the Oxidative Stress-Induced Inflammatory Pathways in Pancreatic Hamster Cells

**DOI:** 10.1155/2016/5843615

**Published:** 2016-03-13

**Authors:** Sher Zaman Safi, Kalaivani Batumalaie, Rajes Qvist, Kamaruddin Mohd Yusof, Ikram Shah Ismail

**Affiliations:** ^1^Faculty of Medicine, Department of Medicine, University of Malaya, 50603 Kuala Lumpur, Malaysia; ^2^Department of Molecular Biology and Genetics, Faculty of Arts and Science, Canik Basari University, Samsun, Turkey

## Abstract

*Purpose.* Type 2 diabetes consists of progressive hyperglycemia and insulin resistance, which could result from glucose toxicity, inflammatory cytokines, and oxidative stress. In the present study we investigated the effect of Gelam honey and quercetin on the oxidative stress-induced inflammatory pathways and the proinflammatory cytokines.* Methods.* HIT-T15 cells were cultured and preincubated with the extract of Gelam honey (20, 40, 60, and 80 *μ*g/mL), as well as quercetin (20, 40, 60, and 80 *μ*M), prior to stimulation by 20 and 50 mM glucose.* Results.* HIT-T15 cells cultured under hyperglycemic condition showed a significant increase in the inflammatory pathways by phosphorylating JNK, IKK-*β*, and IRS-1 at Ser307 (*p* < 0.05). There was a significant decrease in the phosphorylation of Akt at Ser473 (*p* < 0.05). Pretreatment with Gelam honey and quercetin reduced the expression of phosphorylated JNK, IKK-*β*, and IRS-1, thereby significantly reducing the expression of proinflammatory cytokines like TNF-*α*, IL-6, and IL-1*β* (*p* < 0.05). At the same time there was a significant increase in the phosphorylated Akt showing the protective effects against inflammation and insulin resistance (*p* < 0.05). In conclusion, our data suggest the potential use of the extract from Gelam honey and quercetin in modulating the inflammation induced insulin signaling pathways.

## 1. Research Background

Diabetes is one of the most common noncommunicable diseases affecting millions of people globally [1, 2]. It is one of the most challenging health problems in many developing and industrialized countries and the exact cause is unknown. One of the foremost challenges we now face is to account mechanistically not only for the definition of hyperglycemia, but also for other physiological and biochemical abnormalities, which are the characteristics of the disease [3, 4].

Type 2 diabetes consists of progressive hyperglycemia, insulin resistance, and pancreatic *β* cell failure. The pathogenesis of type 2 diabetes is complex and in most instances clearly requires defects in both *β* cell function and insulin sensitivity, and together both of these abnormalities bring about hyperglycemia. Type 2 diabetes has a metabolic milieu, which is characterized by insulin resistance and chronic acute inflammation. In the last decade, a great deal of attention has been focused on the understanding of insulin resistance which is an important contributor to the development and maintenance of hyperglycemia in type 2 diabetes [5, 6].

Β cell dysfunction can result from glucose toxicity, inflammatory cytokines, oxidative stress, or lipotoxicity in the presence of excess glucose. Oxidative stress through the production of reactive oxygen species (ROS) has been proposed as the root cause underlying the development of insulin resistance, *β* cell dysfunction, impaired glucose tolerance, and type 2 diabetes mellitus [3].

Oxidative stress is induced by reactive oxygen and nitrogen species produced by several biochemical pathways associated with hyperglycemia (glucose autooxidation, polyol pathway, prostanoid synthesis, and protein glycation) and is critically involved in the impairment of *β* cell function during the development of type 2 diabetes [7, 8]. Reactive oxygen species can function as signaling molecules to activate a number of stress sensitive pathways and inflammatory pathways leading to the production of inflammatory markers such as IL-6 and TNF-*α* that impair insulin signaling through serine phosphorylation of IRS-1 [9].

Insulin is a pleiotropic hormone which has many functions that are exerted across a variety of insulin target tissues, through several intracellular signaling cascades. Insulin substrate (IRS-1) mediated insulin signaling regulates peripheral insulin action as well as pancreatic *β* cell function by regulating proliferation, survival, and insulin secretion. The defects in insulin signaling pathway mainly involve IRS-1. Activation of the insulin receptor leads to tyrosine phosphorylation of IRS-1, thereby initiating signal transduction. Insulin receptor tyrosine kinases can signal through the phosphatidylinositol 3-kinase (PI3K) pathway, which is mainly responsible for the metabolic actions of insulin. The major target of PI3K is the serine threonine kinase Akt. It has been shown that the Akt dependent mechanism in the distal events of exocytosis is responsible for the defect in insulin secretion [7, 10].

This transient exposure of *β* cells to oxidative stress interrupts the normal coupling of glucose metabolism to insulin secretion by activating stress signaling pathways and the inflammatory pathways [11]. Despite the multiplicity of inflammatory pathways, the development of insulin resistance is often due to the activation of jun-N-terminal kinase (JNK) and the IkappaB kinase (IKK-*β*). The JNK and IKK-*β* pathways are activated by ROS and various other factors including the inflammatory cytokines such as TNF-*α*, IL-6, and IL-1beta which are involved in the development of insulin resistance found in type 2 diabetes [12].

Reactive oxygen species and mitochondrial stress-induced [13] activation of JNK and IKK-*β* promotes the phosphorylation of IRS-1 at serine sites that negatively regulate normal signaling through the insulin receptor/IRS-1 axis, as seen in the phosphorylation of IRS-1 at serine 307 (Ser307). It has been reported that serine phosphorylation of insulin receptor-1 (IRS-1) inhibits insulin stimulated tyrosine phosphorylation of IRS-1 leading to an increase in insulin resistance [10]. Therefore, one of the causes leading to a defect in insulin signaling can be attributed to serine phosphorylation of IRS-1 at serine 307 residues which activate the jun-N-terminal kinase (JNK) and IKK-*β*, thus providing a plausible mechanistic link between inflammation and insulin resistance [14].

Recently, protein kinase B (PKB and Akt) has been shown to function in the insulin signaling cascade by phosphorylating transcription factors which are responsible for the transcription and expression of genes, related to insulin synthesis and secretion. Previous studies have shown that inactivation of Akt can lead to insulin resistance, decreased *β* cell mass, and impaired insulin secretion [15].

Numerous studies have shown that honey has antioxidant and scavenging properties, which prevents the oxidative damage caused by free radicals. The antioxidant property is due to the phenolic compounds which are present in the honey and it has been demonstrated that the biological activities correlate with the phenolic content of the honey [16]. The phenolic content of the Malaysian Gelam honey has been shown to have both anti-inflammatory and antioxidant properties [17]. Quercetin is one of the important components of Gelam honey that may be further elaborated in terms of its function and use as antidiabetic agent.

Therefore, the aim of our present study is to determine the effect of Gelam honey extract and quercetin on the JNK and IKK-*β* inflammatory pathways and IRS-1 serine phosphorylation which causes insulin resistance and the Akt activated insulin signaling pathway, which improves insulin resistance.

## 2. Research Design and Methods

### 2.1. Extraction of Phenolic Compounds from Honey by Solid Phase Extraction (SPE)

Gelam honey samples (Department of Agriculture, Parit Botak, Johor, Malaysia) were subjected to base hydrolysis and extracted with ethyl acetate as described by Wahdan [18] and Seo and Morr [19]. The recovered fractions were combined and dried under nitrogen gas.

### 2.2. Determination of the Phenolic Content

Phenolic compounds from the extract were assayed using Folin-Ciocalteu assay [20]. Briefly the extract (1 mL) was added to 10% Folin-Ciocalteu reagent (Sigma F9252) and 0.5% sodium carbonate. The contents were thoroughly mixed and allowed to stand for 2 hours. The absorbance of the blue color that developed after 2 hours was read at 765 nm. Results were expressed in micrograms of Gallic acid per gram of the extract, using a standard curve generated with Gallic acid (Sigma G7384).

### 2.3. Determination of the Flavonoid Content

The total flavonoid (TF) content was determined spectrophotometrically. Briefly 1 mL of honey extract or a standard solution of quercetin (Sigma Q4951) (10, 50, 100, 150, 200, and 250 *μ*g/mL) in distilled water was added to a 10 mL volumetric flask containing 4 mL of double distilled water, 300 *μ*L of NaNO_2_ (5%, v/v), and 300 *μ*L of 10% AlCl_3_. The solution was allowed to stand at room temperature in the dark for 30 minutes and the absorbance was read at 430 nm. The TF content was determined using the standard curve of quercetin (*μ*g/mL) and was expressed as *μ*g of quercetin equivalents in 1 g of extract.

### 2.4. Cell Culture

HIT-T15 cells were cultured according to the instructions provided by ATCC (CRL-1777). On arrival the cells were cultured immediately in T-25 cm flask in the F12K medium (ATCC 30-2004) supplemented with 10% FBS and 1% penicillin and streptomycin at 37°C (5% CO_2_ in air). Cells (3rd passage) were trypsinized and subcultured, following which they were incubated for 5 days until they reached 80% confluency.

### 2.5. Treatment of HIT-T15 Cells with Quercetin and Gelam Honey Extract

HIT-T15 cells (5 × 10^5^) were pretreated with Gelam honey extract (20, 40, 60, and 80 *μ*g/mL) and quercetin (20, 40, 60, and 80 *μ*M) for 24 hours, after which the medium was replaced with fresh medium. Following this glucose (Sigma G8769) (20 or 50 mM) was added and the cells were incubated for another 24 hours.

### 2.6. Cell Lysate Preparation

After treatment the cells were washed twice in PBS and lysed in mammalian cell lysis buffer (Sigma MCL-1 which was supplemented with protease and phosphatase inhibitors. Insoluble materials were eliminated by centrifugation (12,000 ×g, 10 min, 4°C), and the protein concentration in the supernatant was determined by Bradford assay (Bio-Rad Laboratories).

### 2.7. Western Blot Analysis

Thirty micrograms of protein extracts was loaded on 10% SDS-polyacrylamide gel and transferred to activated nitrocellulose membrane. The membranes were blocked with tris-buffered saline (TBS) containing 5% nonfat milk and incubated with pAkt (Ser473), pIRS-1 (Ser307), p-JNK, p-IKK-*α*/*β*, IL-6, IL-1*β*, and TNF alpha primary antibodies (obtained from Santa Cruz Laboratories) overnight at 4°C with a concentration of 1 : 1000. *β*-actin was used as a loading control. After 3 times extensive washes in TBS (for 20–30 m each), membranes were incubated for 1 h at room temperature with the appropriate horseradish peroxidase-conjugated secondary antibodies. We use the concentration of 1 : 5000 for secondary antibodies. Then we visualized the film using chemiluminescence substrate according to the manufacturer's instructions (Amersham Life Sciences, Little Chalfont, UK). Quantitative analysis of the protein content was performed by Gel Documentation System (Biospectrum 410, UVP).

### 2.8. Statistical Analyses

Data were analyzed with one-way ANOVA using SPSS version 16.0 software. The results were expressed as the mean ± standard deviation value. ^*∗*^ *p* < 0.05 was considered to be statistically significant.

## 3. Results

### 3.1. Total Phenolic and Flavonoid Content

10 g of liquefied fresh Malaysian Gelam honey (*Apis mellifera*) was extracted using ethyl acetate and the extract was found to contain 52 *μ*g of Gallic acid per gram of extract of total phenolic content and 6.92 *μ*g of quercetin per gram of extract of total flavonoid content.

In our previous paper [21] we demonstrated the presence of ROS by using 2′7′-dichlorodihydrofluorescein diacetate (DCFH-DA) reagent under hyperglycemic conditions and by the measurement of intracellular ROS in individual cells by image analysis. Our data showed the antioxidant effect of Gelam honey extract and the flavonoid compounds quercetin, luteolin, and chrysin in the cultured cells by significantly inhibiting the production of ROS in a dose dependent manner. Again our data showed the reduction of ROS in the single cells in a dose dependent manner after pretreatment with the honey extract and the flavonoids.

### 3.2. Effect of Pretreatment with Quercetin and Gelam Honey Extract on Phospho-JNK Expression under Normal and Hyperglycemic Conditions

**Figure 1:**
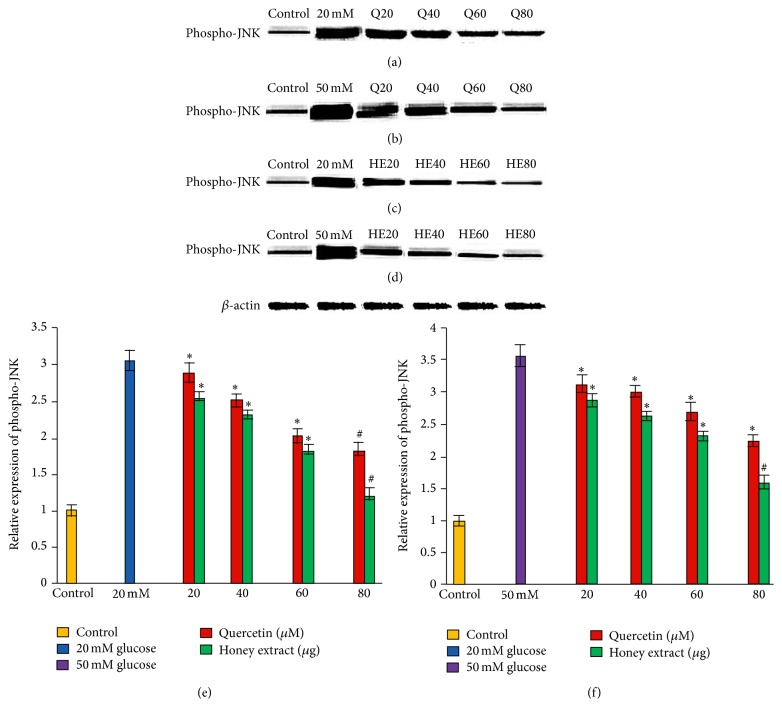
Effect of quercetin and Gelam honey extract on phospho-JNK expression. Quantitative analysis and representative western blot analysis of phospho-JNK in HIT-T15 cells pretreated with quercetin and honey extract in cells cultured in 20 mM ((a), (c), and (e)) and 50 mM ((b), (d), and (f)) glucose. The results were normalized with *β*-actin antibody. Data were analyzed with one-way ANOVA using SPSS version 16.0 software and are presented as the mean ± standard deviation and represent the mean of three different experiments. (e) ^*∗*^ *p* < 0.05 and ^#^ *p* < 0.005, quercetin and honey extract treated compared to the 20 mM glucose alone. (f) ^*∗*^ *p* < 0.05 and ^#^ *p* < 0.005, quercetin and honey extract treated compared to the 50 mM glucose alone.

HIT-T15 cells were pretreated with the quercetin at concentrations of 20, 40, 60, and 80 *μ*M and Gelam honey extract at concentrations of 20, 40, 60, and 80 *μ*g/mL for 24 hours, following which they were cultured with 20 mM (Figures 1(a), 1(c), and 1(e)) or 50 mM (Figures 1(b), 1(d), and 1(f)) glucose to determine the phosphorylation of JNK. The data revealed that exposure of HIT-T15 cells to 20 and 50 mM glucose significantly increased the level of phospho-JNK expression compared to control. Pretreatment with quercetin and Gelam honey extract significantly (*p* < 0.05) reduced the ROS induced expression of phospho-JNK under 20 mM glucose (Figures 1(a), 1(c), and 1(e)) by 60% and 42% in a dose dependent manner. Pretreatment with quercetin and Gelam honey extract reduced the expression of phospho-JNK significantly (*p* < 0.05) by 64% and 50%, respectively, compared to the cells that were cultured with 50 mM glucose (Figures 1(b), 1(d), and 1(f)) alone. The phospho-JNK protein levels from each sample were normalized to their respective *β*-actin protein amounts (^*∗*^ *p* < 0.05 and ^#^ *p* < 0.005 versus glucose treated group) (Figure 1).

### 3.3. Effect of Pretreatment with Quercetin and Gelam Honey Extract on Phospho-IKK-*β* Expression under Normal and Hyperglycemic Conditions

**Figure 2:**
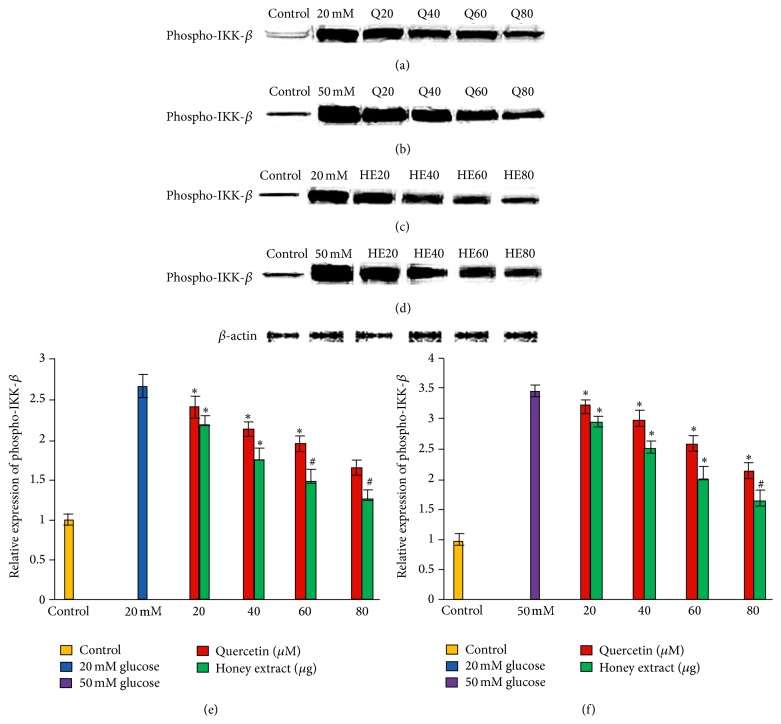
Effect of quercetin and Gelam honey extract on phospho-IKK-*β* expression. Quantitative analysis and representative western blot analysis of phospho-IKK-*β* in HIT-T15 cells pretreated with quercetin and honey extract in cells cultured in 20 mM ((a), (c), and (e)) and 50 mM ((b), (d), and (f)) glucose. The results were normalized with *β*-actin antibody. Data were analyzed with one-way ANOVA using SPSS version 16.0 software and are presented as the mean ± standard deviation and represent the mean of three different experiments. (e) ^*∗*^ *p* < 0.05 and ^#^ *p* < 0.005, quercetin and honey extract treated compared to the 20 mM glucose alone. (f) ^*∗*^ *p* < 0.05 and ^#^ *p* < 0.005, quercetin and honey extract treated compared to the 50 mM glucose alone.

HIT-T15 cells were pretreated with the quercetin at concentrations of 20, 40, 60, and 80 *μ*M and Gelam honey extract at concentrations of 20, 40, 60, and 80 *μ*g/mL for 24 hours, following which they were cultured with 20 mM (Figures 2(a), 2(c), and 2(e)) or 50 mM (Figures 2(b), 2(d), and 2(f)) glucose to determine the phosphorylation of IKK-*β*. The phosphorylation of IKK-*β* was increased in HIT-T15 cells treated with 20 mM and 50 mM glucose alone as compared with control. Pretreatment with the quercetin and Gelam honey extract showed 55% and 44% decrease (*p* < 0.05) in the expression of phosphorylated IKK-*β* in a dose dependent manner in the cells that were cultured in 20 mM (Figures 2(a), 2(c), and 2(e)) glucose. Pretreatment with quercetin and Gelam honey extract significantly (*p* < 0.05) decreased the expression of phosphorylated IKK-*β* under 50 mM glucose (Figures 2(b), 2(d), and 2(f)) by 60% and 47% in a dose dependent manner. The phosphorylated IKK-*β* protein levels from each sample were normalized to their respective *β*-actin protein amounts (^*∗*^ *p* < 0.05 and ^#^ *p* < 0.005 versus glucose treated group) (Figure 2).

### 3.4. Effect of Pretreatment with Quercetin and Gelam Honey Extract on IL-6 Expression under Normal and Hyperglycemic Conditions

**Figure 3:**
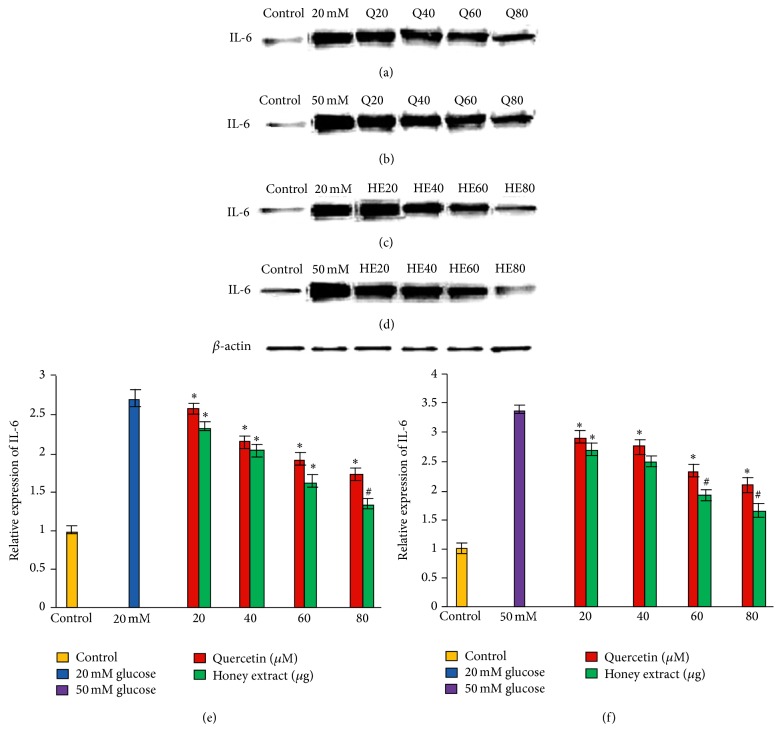
Effect of quercetin and Gelam honey extract on IL-6 expression. Quantitative analysis and representative western blot analysis of IL-6 in HIT-T15 cells pretreated with quercetin and honey extract in cells cultured in 20 mM ((a), (c), and (e)) and 50 mM ((b), (d), and (f)) glucose. The results were normalized with *β*-actin antibody. Data were analyzed with one-way ANOVA using SPSS version 16.0 software and are presented as the mean ± standard deviation and represent the mean of three different experiments. (e) ^*∗*^ *p* < 0.05 and ^#^ *p* < 0.005, quercetin and honey extract treated compared to the 20 mM glucose alone. (f) ^*∗*^ *p* < 0.05 and ^#^ *p* < 0.005, quercetin and honey extract treated compared to the 50 mM glucose alone.

HIT-T15 cells were pretreated with the quercetin at concentrations of 20, 40, 60, and 80 *μ*M and Gelam honey extract at concentrations of 20, 40, 60, and 80 *μ*g/mL for 24 hours, following which they were cultured in 20 mM (Figures 3(a), 3(c), and 3(e)) or 50 mM (Figures 3(b), 3(d), and 3(f)) glucose to determine the expression of IL-6. The expression was increased in HIT-T15 cells treated with 20 mM and 50 mM glucose alone as compared with control. Pretreatment with the quercetin and Gelam honey extract showed 52% and 40% decrease (*p* < 0.05) in the expression of IL-6 in a dose dependent manner in the cells that were cultured in 20 mM glucose (Figures 3(a), 3(c), and 3(e)). Pretreatment with quercetin and Gelam honey extract significantly (*p* < 0.05) decreased the expression of IL-6 under 50 mM glucose (Figures 3(b), 3(d), and 3(f)) by 60% and 47% in a dose dependent manner. The IL-6 protein levels from each sample were normalized to their respective *β*-actin protein amounts (^*∗*^ *p* < 0.05 and ^#^ *p* < 0.005 versus glucose treated group).

### 3.5. Effect of Pretreatment with Quercetin and Gelam Honey Extract on IL-1*β* Expression under Normal and Hyperglycemic Conditions

**Figure 4:**
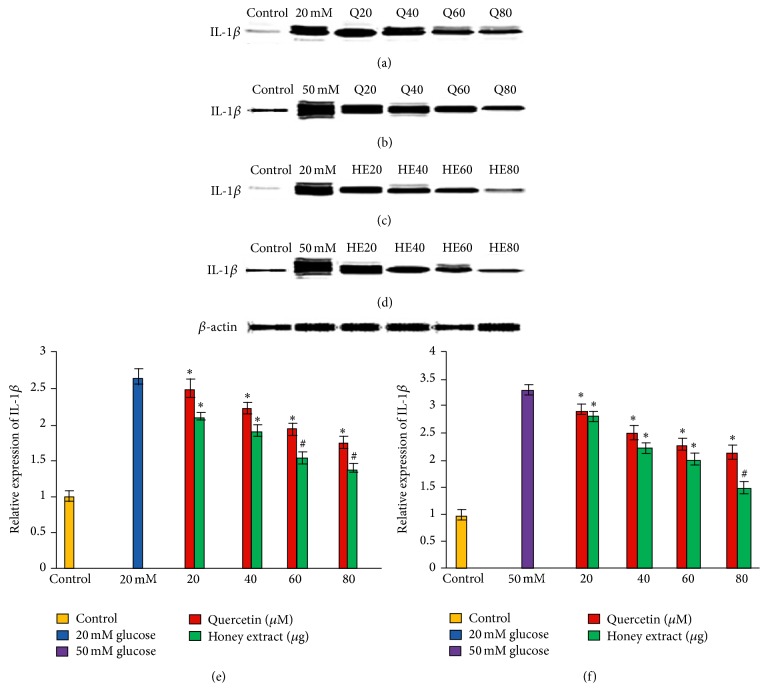
Effect of quercetin and Gelam honey extract on IL-1*β* expression. Quantitative analysis and representative western blot analysis of IL-1*β* in HIT-T15 cells pretreated with quercetin and honey extract in cells cultured in 20 mM ((a), (c), and (e)) and 50 mM ((b), (d), and (f)) glucose. The results were normalized with *β*-actin antibody. Data were analyzed with one-way ANOVA using SPSS version 16.0 software and are presented as the mean ± standard deviation and represent the mean of three different experiments. (e) ^*∗*^ *p* < 0.05 and ^#^ *p* < 0.005, quercetin and honey extract treated compared to the 20 mM glucose alone. (f) ^*∗*^ *p* < 0.05 and ^#^ *p* < 0.005 quercetin and honey extract treated compared to the 50 mM glucose alone.

HIT-T15 cells were pretreated with the quercetin at concentrations of 20, 40, 60, and 80 *μ*M and Gelam honey extract at concentrations of 20, 40, 60, and 80 *μ*g/mL for 24 hours, following which they were then cultured with 20 or 50 mM glucose to determine the expression of IL-1*β*. IL-1*β* in HIT-T15 cells was markedly increased following 20 mM (Figures 4(a), 4(c), and 4(e)) and 50 mM (Figures 4(b), 4(d), and 4(f)) glucose treatment, but the trend was reversed after pretreatment with quercetin and Gelam honey extract. Pretreatment of the cells with different concentration of quercetin and honey extract for 24 hours significantly reduced the expression of IL-1*β* up to 55% and 42%, respectively, compared to the cells that were cultured alone with 20 mM glucose (Figures 4(a), 4(c), and 4(e)). On the other hand, pretreatment with quercetin and honey extract reduced the expression of IL-1*β* significantly (*p* < 0.05) up to 63% and 54%, respectively, compared to the cells that were cultured alone with 50 mM glucose (Figures 4(b), 4(d), and 4(f)). The IL-1*β* protein levels from each sample were normalized to their respective *β*-actin protein amounts (^*∗*^ *p* < 0.05 and ^#^ *p* < 0.005 versus glucose treated group), Figure 3.

### 3.6. Effect of Pretreatment with Quercetin and Gelam Honey Extract on TNF Alpha Expression under Normal and Hyperglycemic Conditions

**Figure 5:**
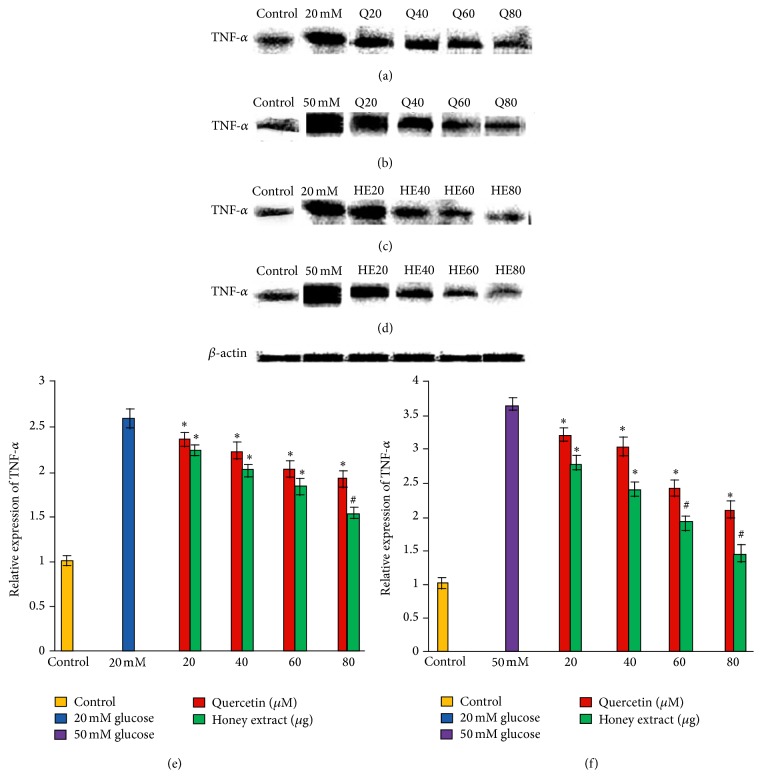
Effect of quercetin and Gelam honey extract on TNF alpha expression. Quantitative analysis and representative western blot analysis of TNF alpha in HIT-T15 cells pretreated with quercetin and honey extract in cells cultured in 20 mM ((a), (c), and (e)) and 50 mM ((b), (d), and (f)) glucose. The results were normalized with *β*-actin antibody. Data were analyzed with one-way ANOVA using SPSS version 16.0 software and are presented as the mean ± standard deviation and represent the mean of three different experiments. (e) ^*∗*^ *p* < 0.05 and ^#^ *p* < 0.005, quercetin and honey extract treated compared to the 20 mM glucose alone. (f) ^*∗*^ *p* < 0.05 and ^#^ *p* < 0.005, quercetin and honey extract treated compared to the 50 mM glucose alone.

HIT-T15 cells were pretreated with the quercetin at concentrations of 20, 40, 60, and 80 *μ*M and Gelam honey extract at concentrations of 20, 40, 60, and 80 *μ*g/mL for 24 hours, following which they were then cultured with 20 mM (Figures 5(a), 5(c), and 5(e)) or 50 mM (Figures 5(b), 5(d), and 5(f)) glucose to determine the TNF alpha. The TNF alpha was increased in HIT-T15 cells treated with 20 mM and 50 mM glucose alone as compared with control. Pretreatment with the quercetin and Gelam honey extract showed 43% and 55% decrease (*p* < 0.05) in the expression of TNF alpha in a dose dependent manner in the cells that were cultured in 20 mM glucose (Figures 5(a), 5(c), and 5(e)). Pretreatment with quercetin and Gelam honey extract significantly (*p* < 0.05) decreased the expression of TNF alpha in a dose dependent manner, in the cells cultured with 50 mM glucose (Figures 5(b), 5(d), and 5(f)) by 52% and 66%. The TNF alpha protein levels from each sample were normalized to their respective *β*-actin protein amounts (^*∗*^ *p* < 0.05 and ^#^ *p* < 0.005 versus glucose treated group) (Figure 5).

### 3.7. Effect of Pretreatment with Quercetin and Gelam Honey Extract on pIRS-1 (Ser307) Expression under Normal and Hyperglycemic Conditions

**Figure 6:**
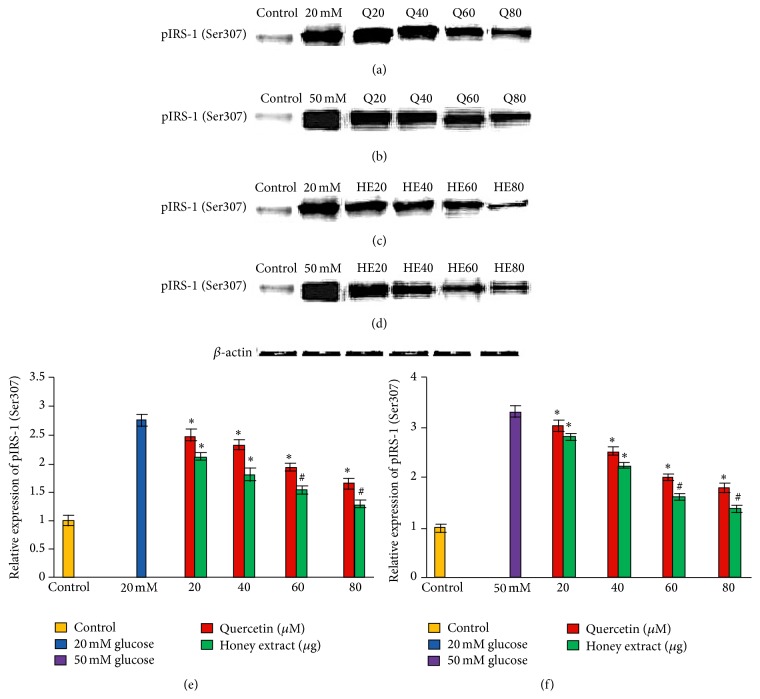
Effect of quercetin on pIRS-1 (Ser307) expression. Quantitative analysis and representative western blot analysis of pIRS-1 (Ser307) in HIT-T15 cells pretreated with quercetin and Gelam honey extract in cells cultured in 20 mM ((a), (c), and (e)) and 50 mM ((b), (d), and (f)) glucose. The results were normalized with *β*-actin antibody. Data were analyzed with one-way ANOVA using SPSS version 16.0 software and are presented as the mean ± standard deviation and represent the mean of three different experiments. (e) ^*∗*^ *p* < 0.05 and ^#^ *p* < 0.005, quercetin and honey extract treated compared to the 20 mM glucose alone. (f) ^*∗*^ *p* < 0.05 and ^#^ *p* < 0.005 quercetin and honey extract treated compared to the 50 mM glucose alone.

HIT-T15 cells were pretreated with the quercetin at concentrations of 20, 40, 60, and 80 *μ*M and Gelam honey extract at concentrations of 20, 40, 60, and 80 *μ*g/mL for 24 hours, following which they were then cultured with 20 mM (Figures 6(a), 6(c), and 6(e)) or 50 mM (Figures 6(b), 6(d), and 6(f)) glucose to determine the phosphorylation of IRS-1 (Ser307). The phosphorylation of IRS-1 (Ser307) was increased in HIT-T15 cells treated with 20 mM and 50 mM glucose alone as compared with control. Pretreatment with the quercetin and Gelam honey extract showed 49% and 55% decrease (*p* < 0.05) in the expression of pIRS-1 (Ser307) in a dose dependent manner in the cells that were cultured in 20 mM glucose (Figures 6(a), 6(c), and 6(e)). Pretreatment with quercetin and Gelam honey extract significantly (*p* < 0.05) decreased the expression of pIRS-1 (Ser307) in cells that were cultured in 50 mM glucose (Figures 6(b), 6(d), and 6(f)) by 43% and 60% in a dose dependent manner. The pIRS-1 protein levels from each sample were normalized to their respective *β*-actin protein amounts (^*∗*^ *p* < 0.05 and ^#^ *p* < 0.005 versus glucose treated group) (Figure 6).

### 3.8. Effect of Pretreatment with Quercetin and Gelam Honey Extract on pAkt (Ser473) Expression under Normal and Hyperglycemic Conditions

**Figure 7:**
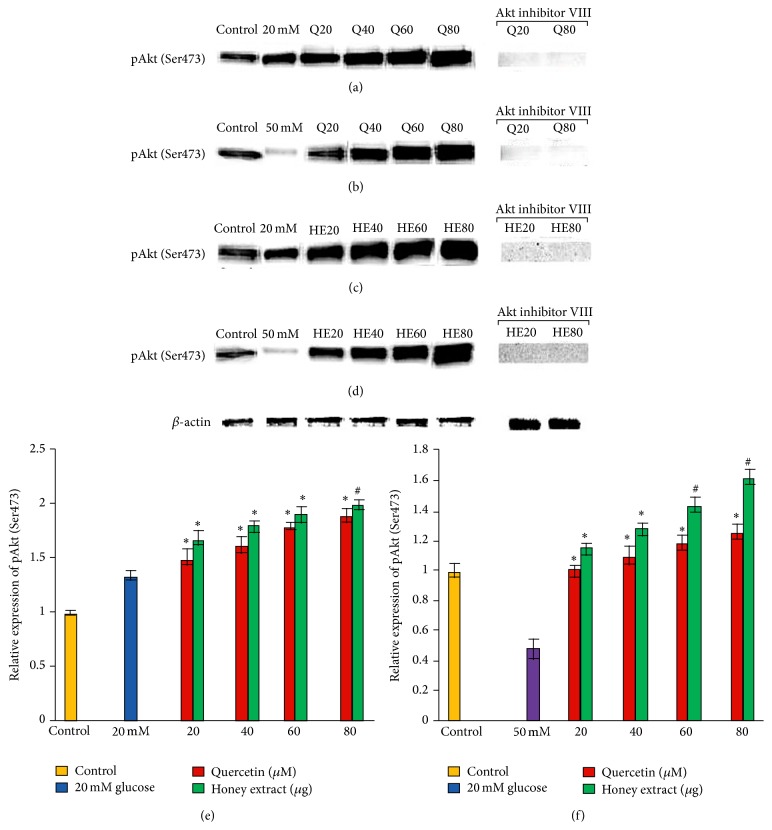
Effect of quercetin and Gelam honey extract on pAkt (Ser473) expression. Quantitative analysis and representative western blot analysis of pAkt (Ser473) in HIT-T15 cells pretreated with quercetin and honey extract in cells cultured in 20 mM ((a), (c), and (e)) and 50 mM ((b), (d), and (f)) glucose. An increase in the level of pAkt (Ser473) was observed after pretreatment with quercetin and honey extract. Akt inhibitor VIII prevented the expression of Akt Ser473 phosphorylation induced by quercetin and honey extract. The results were normalized with *β*-actin antibody. Data were analyzed with one-way ANOVA using SPSS version 16.0 software and are presented as the mean ± standard deviation and represent the mean of three different experiments. (e) ^*∗*^ *p* < 0.05 and ^#^ *p* < 0.005, quercetin and honey extract treated compared to the 20 mM glucose alone. (f) ^*∗*^ *p* < 0.05 and ^#^ *p* < 0.005, quercetin and honey extract treated compared to the 50 mM glucose alone.

HIT-T15 cells were pretreated with the quercetin at concentrations of 20, 40, 60, and 80 *μ*M and Gelam honey extract at concentrations of 20, 40, 60, and 80 *μ*g/mL for 24 hours, following which they were then cultured with 20 or 50 mM glucose to determine the phosphorylation of Akt (Ser473). There was no change in the Akt (Ser473) phosphorylation in HIT-T15 cells when treated with 20 mM glucose (Figures 7(a), 7(c), and 7(e)) but it was markedly reduced when the cells were treated with 50 mM (Figures 7(b), 7(d), and 7(f)) glucose. Pretreatment of the cells with different concentration of quercetin and honey extract for 24 hours significantly increased the expression of pAkt (Ser473) up to 38% and 70%, respectively, compared to the cells that were cultured alone with 20 mM glucose (Figures 7(a), 7(c), and 7(e)). On the other hand, pretreatment with quercetin and honey extract increased the expression of pAkt (Ser473) significantly (*p* < 0.05) up to 30% and 54%, respectively, compared to the cells that were cultured alone with 50 mM glucose (Figures 7(b), 7(d), and 7(f)). The cells that were exposed to Akt inhibitor VIII prevented the quercetin and honey extract induced Akt Ser473 phosphorylation (Figure 7). The pAkt protein levels from each sample were normalized to their respective *β*-actin protein amounts (^*∗*^ *p* < 0.05 and ^#^ *p* < 0.005 versus glucose treated group) (Figure 7).

## 4. Discussion

In our previous study we investigated the antioxidant effect of the Malaysian Gelam honey and some of its flavonoid components (chrysin, luteolin, and quercetin) individually, on pancreatic hamster cells (HIT-T15) cultured under hyperglycemic conditions. Our data demonstrated that the cultured cells, pretreated with the extract of the Gelam honey and the different flavonoid components (quercetin, luteolin, and chrysin) at varying concentrations for 24 hours, protected the *β* cell from oxidative damage caused by ROS induced by hyperglycemia [21]. Furthermore, we investigated the effect of Gelam honey extract and quercetin on the stress activated NF-*κ*B and MAPK pathways and IRS-1 serine phosphorylation causing insulin resistance and the Akt activated insulin signaling pathway, causing increase in insulin content. Our data demonstrated that the Gelam honey extract and quercetin had the best protective effect against hyperglycemia induced oxidative stress by improving the insulin content and insulin resistance [22].

The floral source of Gelam honey is* Melaleuca cajuputi*. This was the only source of Gelam honey that was used in Kamaruddin et al. laboratory and different students worked on the different aspects of this honey. They were the first to establish the detection of the phenolic compounds in Gelam honey found in Malaysia using HPLC [23]. Chromatogram of phenolic acid and flavonoids detected in Malaysian Gelam honey, using HPLC-UV absorption (1) 290 nm and (2) 340 nm, is shown in the paper of the same group [24]. In our previous study we used three different flavonoids: chrysin, luteolin, and quercetin [21, 22]. This shows that the quercetin was the most effective amongst the three flavonoids. The work on caffeic acid on the same Gelam honey has already been published by the above-mentioned group [25, 26].

Therefore, in our present study, we determined the effect of Gelam honey extract and quercetin on the oxidative stress activated JNK and IKK-*β* inflammatory pathways and IRS-1 serine phosphorylation causing insulin resistance and the Akt activated insulin signaling pathway, which attenuates the inflammation, thereby increasing insulin sensitivity.

Several studies have demonstrated that flavonoids may reduce hyperglycemia and exert protective effects against nonenzymatic glycation of proteins in animals [27, 28]. The flavonoid quercetin in particular has been reported to have anti-inflammatory properties in diabetic animal models, by protecting the retinal cell apoptosis in diabetes [29]. Under diabetic conditions, hyperglycemia induced ROS and inflammatory cytokines presumably lead to the activation of the JNK and IKK-*β* pathways. It has been shown that IKK and JNK control the major inflammatory response pathways in hyperglycemia and that they are activated by oxidative stress [30, 31].

Yuan et al. [32] demonstrated that reduced signaling through the IKK-*β* pathway inhibition by sodium salicylate in obese mice is accompanied by improved insulin sensitivity. Again the study showed that phosphorylation of IKK-*β* was increased in diabetes, but the blockade of NF-*κ*B, oxidative stress, and the genetic deletion of TNF-*α* attenuated phosphorylation of IKK-*β*, suggesting that the phosphorylation of IKK-*β* is increased in diabetic mice by the activation of oxidative stress and TNF-*α* [33].

The increase in free fatty acids, inflammatory cytokines, and oxidative stress under diabetic conditions activates the JNK pathway which causes insulin resistance and pancreatic *β* cell dysfunction [34]. It has been reported that the JNK pathway was abnormally activated in the liver, muscle, and adipose tissue in obese type 2 diabetic mice and that the insulin resistance was substantially reduced when the mice were homozygous for a targeted mutation in the JNK1 gene.

Our data showed that exposure of HIT-T15 cells to 20 and 50 mM glucose caused a significant increase in the level of phosphorylated JNK (Figure 1) and phosphorylated IKK-*β* (Figure 2) expressions compared to the control. Pretreatment with quercetin and Gelam honey extract significantly (*p* < 0.05) reduced the ROS induced inflammatory pathway of phosphorylated JNK (Figure 1) and phosphorylated IKK-*β* (Figure 2) when treated with 20 and 50 mM glucose. This gives further support to the fact that oxidative stress induced by high glucose activates the major inflammatory pathways.

It has been shown that hyperglycemia is proinflammatory and is normally restrained by the anti-inflammatory effect of insulin secreted in response to that stimulus [35]. Hyperglycemia induced by glucose clamping in normal subjects showed a significant increase in the circulating levels of interleukin- (IL-) 6, IL-18, Il-1*β*, and tumor necrosis factor-*α* (TNF-*α*). These effects were more sustained in patients with impaired glucose tolerance as well as in those after consecutive pulses of intravenous glucose, which were annulled by glutathione, implicating an oxidative mechanism [31]. Interestingly enough, these cytokines have been implicated in insulin resistance (TNF-*α*, IL-6, and IL-1*β*), atherosclerotic plaque destabilization (IL-18), and future cardiovascular events (IL-6, IL-18, IL-1*β*, and TNF-*α*). So, an increased oxidative stress may be a likely mechanism linking stress hyperglycemia to increased inflammatory cytokine production [31, 34]. Several cross-sectional studies showed that insulin resistance and type 2 diabetes are associated with higher levels of C-reactive protein (CRP), interleukin-6 (IL-6), interleukin-1*β*, and tumor necrosis factor-*α* (TNF-*α*), which are markers of subclinical systemic inflammation [35]. Furthermore, various longitudinal studies have shown that elevated levels of CRP, IL-1*β*, IL-6, and TNF-*α* predict the development of type 2 diabetes [35].

Our data supported the above statement by showing that TNF-*α*, IL-6, and IL-1*β* expressions (Figures 3, 4, and 5) in HIT-T15 cells were markedly increased following 20 and 50 mM glucose treatment but were reversed after pretreatment with quercetin and Gelam honey extract. In addition, our data showed that the expression of TNF-*α*, IL-6, and IL-1*β* (Figures 3, 4, and 5) was decreased.

Our previous data demonstrated that Akt (Ser473) phosphorylation was increased after pretreatment with quercetin and honey extract (REF). Effect of Gelam honey on the oxidative stress induced signaling pathways in pancreatic hamster cells [22].

In the year 2001 it was reported that mice lacking the pAkt (Ser473) protein were insulin resistant, with impaired insulin secretion [36]. It was shown that Akt (Ser473) is necessary for normal pancreatic *β* cell function including a novel regulatory role for Akt signaling in insulin secretion [37]. Several studies reported that a decrease in insulin secretion and insulin resistance induced by hyperglycemia has been associated with decreased Akt activity [38–41].

In our previous papers [21, 22] we showed that the cells cultured under 20 mM glucose did not show any changes at the level of the Akt phosphorylation while under 50 mM the Akt (serine 473) phosphorylation was reduced significantly. The phosphorylation was increased significantly after pretreatment with quercetin and honey extract. Akt inhibitor VIII prevented the expression of Akt Ser473 phosphorylation induced by quercetin and honey extract. When we studied the insulin content our data demonstrated that there was a significant increase in the insulin content (*p* < 0.05) when the cells cultured under 20 mM glucose were pretreated with quercetin and honey extract, but there was a significant decrease in the insulin content (*p* < 0.05) when the cells were treated with Akt inhibitor VIII, before pretreating with quercetin and Gelam honey extract when the cells were cultured in 50 mM glucose, and there was a significant increase in the insulin content (*p* < 0.05 and *p* < 0.005) when the cells were pretreated with quercetin and Gelam honey extract. A significant decrease was seen (*p* < 0.05 and *p* < 0.005) when the cells were treated with Akt inhibitor VIII before pretreating with quercetin and Gelam honey extract.

Thus, our data demonstrated the protective effects of Akt against *β* cell dysfunction and insulin resistance, which was further supported by the fact that Akt inhibitor increased the insulin resistance by decreasing the quantity of insulin secreted.

It has been shown that activation of the IKK-*β* and JNK pathways increases IRS-1 serine phosphorylation which leads to suppression of insulin signaling and that suppression of the IKK pathway decreases insulin resistance and ameliorates glucose intolerance in diabetic mice [29]. Our data which supports the above statement showed that phosphorylation of IRS-1 (Ser307) was increased in HIT-T15 cells treated with 20 and 50 mM glucose alone as compared with control. Pretreatment with the quercetin and Gelam honey extract significantly decreased (*p* < 0.05) the expression of pIRS-1 (Ser307) (Figure 6) in a dose dependent manner in the cells that were cultured in 20 and 50 mM glucose (Figure 6). Our data suggest that there is decrease in phosphorylation of IRS-1(Ser307) (Figure 6).

## 5. Conclusion

In conclusion our data suggest the potential use of the extract from Gelam honey in treating diabetes, by modulating the inflammatory pathways. The data provide further support to the role of inflammation in *β* cell dysfunction and insulin resistance. In addition, since stress-induced JNK and IKK pathways are involved in the development of insulin resistance, inflammation, and *β* cell dysfunction, it is possible that such pathways could be a therapeutic target for type 2 diabetes.
